# Automated detection and classification of acute vertebral body fractures using a convolutional neural network on computed tomography

**DOI:** 10.3389/fendo.2023.1132725

**Published:** 2023-03-27

**Authors:** Jianlun Zhang, Feng Liu, Jingxu Xu, Qingqing Zhao, Chencui Huang, Yizhou Yu, Huishu Yuan

**Affiliations:** ^1^ Department of Radiology, Peking University Third Hospital, Beijing, China; ^2^ Deepwise AI Lab, Beijing, China

**Keywords:** osteoporosis, trauma, vertebral fracture (VF), deep learning, fracture detection, fracture classification

## Abstract

**Background:**

Acute vertebral fracture is usually caused by low-energy injury with osteoporosis and high-energy trauma. The AOSpine thoracolumbar spine injury classification system (AO classification) plays an important role in the diagnosis and treatment of the disease. The diagnosis and description of vertebral fractures according to the classification scheme requires a great deal of time and energy for radiologists.

**Purpose:**

To design and validate a multistage deep learning system (multistage AO system) for the automatic detection, localization and classification of acute thoracolumbar vertebral body fractures according to AO classification on computed tomography.

**Materials and Methods:**

The CT images of 1,217 patients who came to our hospital from January 2015 to December 2019 were collected retrospectively. The fractures were marked and classified by 2 junior radiology residents according to the type A standard in the AO classification. Marked fracture sites included the upper endplate, lower endplate and posterior wall. When there were inconsistent opinions on classification labels, the final result was determined by a director radiologist. We integrated different networks into different stages of the overall framework. U-net and a graph convolutional neural network (U-GCN) are used to realize the location and classification of the thoracolumbar spine. Next, a classification network is used to detect whether the thoracolumbar spine has a fracture. In the third stage, we detect fractures in different parts of the thoracolumbar spine by using a multibranch output network and finally obtain the AO types.

**Results:**

The mean age of the patients was 61.87 years with a standard deviation of 17.04 years, consisting of 760 female patients and 457 male patients. On vertebrae level, sensitivity for fracture detection was 95.23% in test dataset, with an accuracy of 97.93% and a specificity of 98.35%. For the classification of vertebral body fractures, the balanced accuracy was 79.56%, with an AUC of 0.904 for type A1, 0.945 for type A2, 0.878 for type A3 and 0.942 for type A4.

**Conclusion:**

The multistage AO system can automatically detect and classify acute vertebral body fractures in the thoracolumbar spine on CT images according to AO classification with high accuracy.

## 1 Introduction

Acute vertebral fracture is a common disease, accounting for approximately 14% of all fractures (1). Acute vertebral fracture is usually caused by low-energy injury with osteoporosis or high-energy trauma and is most common in the thoracolumbar vertebrae (2). Acute vertebral fracture has a significant impact on patient health and causes a considerable economic cost burden.

To assist in the diagnosis and treatment of vertebral fractures, many classification systems for vertebral fractures have been developed (3–6). In 2013, Vaccaro et al. incorporated a modified classification system called the AOSpine thoracolumbar spine injury classification system (7), which is a widely accepted and comprehensive classification system used for clinical practice and research (8, 9). This classification system can guide treatment decisions and help with the formulation of surgical procedures (10–12). In the AO classification system, fractures involving only the vertebral body (i.e., types A1, A2, A3, A4) account for the highest proportion at approximately 70% (13). CT is a vital imaging method for evaluating thoracolumbar fractures, especially in emergency situations (14).

The classification system is complex. The diagnosis and description of vertebral fractures according to the classification scheme require considerable time and energy for radiologists (15). However, rapid and accurate diagnosis is very important for guiding patient treatment decisions (16). Deep learning can help solve these problems.

Deep learning algorithms are powerful algorithms for medical image analysis, such as image classification, object detection, and image segmentation (17, 18). Some studies have applied deep learning to the detection of vertebral fractures on X-ray images, and it can assist in measuring vertebral height loss and identifying fresh fractures (19, 20). There are also some explorations on the use of deep learning in identifying osteoporotic vertebral fractures and differentiating benign and malignant vertebral fractures on CT (21, 22). Some automatic segmentation models depending on deep learning in CT were proposed to increase the segmentation efficiency and improve localization precision (23, 24). Deep learning was also applied to classification of vertebral fracture, but the dataset was limited to young people injured by basketball (25). More studies on fracture location and classification are needed, which is the focus of this article.

The purpose of our study was to design and validate a deep learning system for the automatic detection, localization, and classification of acute vertebral body fractures according to the AOSpine thoracolumbar spine injury classification system on computed tomography.

## 2 Materials and methods

### 2.1 Study subjects

The study was approved by the Ethics Management Committee of the hospital. Our study was performed as a retrospective analysis, and informed consent was waived.

The CT images of patients with acute thoracolumbar vertebral fractures who came to our hospital from January 2015 to December 2019 were retrospectively collected from our PACS system. A fracture was defined as an acute fracture if both conditions were met: (1) the imaging examination was performed within 2 weeks after the injury; (2) CT showed clear and sharp fracture lines in the vertebra, or MRI showed an abnormal signal intensity in the vertebra on fat suppression sequences when it was hard to decide by only CT examination. The inclusion criteria were as follows: (1) acute vertebral fracture and (2) involvement of the thoracolumbar vertebrae. The exclusion criteria were as follows: (1) reported pathological fracture (tumor, infection), (2) a history of surgical intervention on the spine before CT examination, (3) poor image quality, (4) type B1, B2, B3, and C. A total of 838 CT images were finally included. A total of 379 additional fracture-negative CT images were collected from July 2019 to December 2019, which were only used for training the deep-learning models. The inclusion criteria were as follows: (1) reported non-fracture. The exclusion criteria were as follows: (1) reported pathological fracture (tumor, infection), (2) a history of surgical intervention on the spine before CT examination, and (3) poor image quality. All CT images were obtained on two 64-slice spiral CT scanners, both SOMATOM Definition Flash (Siemens AG, Forchheim, Germany), tube voltage: 120 kV, rotation time: 0.5 s, section thickness: 1 mm. Sagittal reformations of the spine were reconstructed with a slice thickness of 3 mm. We divide the training dataset, validation dataset and test dataset according to the ratio of about 7:1:2.

### 2.2 Lesion identification

**Figure 1:**
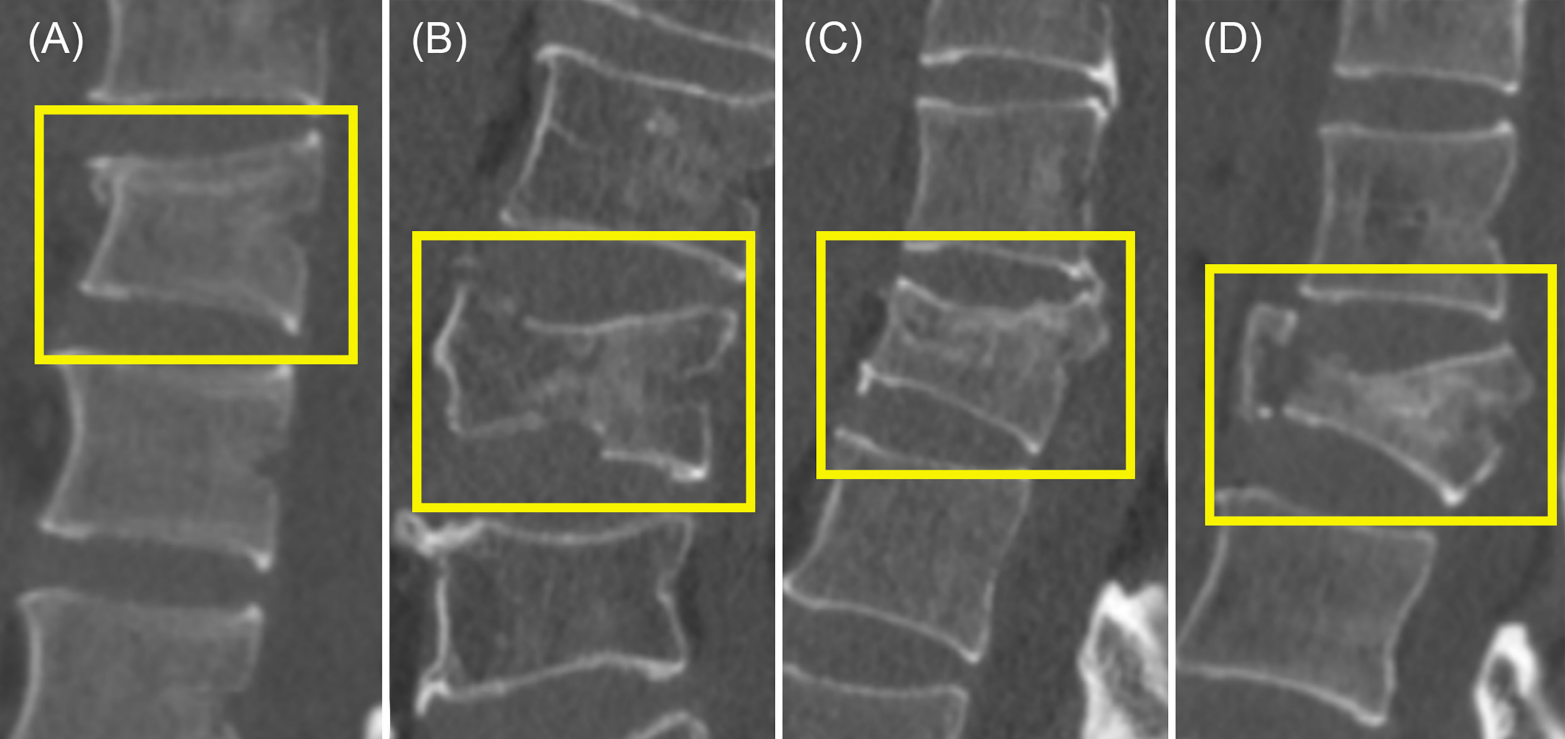
Examples of type A1, A2, A3, and A4. **(A)** Type A1. Only the upper endplate was involved. **(B)** Both the upper endplate and the lower endplate were involved. **(C)** Both the upper endplate and the posterior wall were involved. **(D)** The upper endplate, the lower endplate, and the posterior wall were involved.

Digital Imaging and Communications in Medicine images for each CT examination were downloaded in a noncompressed format. Each CT was labeled by two junior radiology residents. The labels include (1) which vertebral segment the fracture is located in, (2) which part of the vertebral body is involved (upper endplate, lower endplate, posterior wall), and (3) what type of AO classification the fracture belongs to (A1, A2, A3, A4). The classification standards were as follows (7): (1) with any involvement of the posterior wall, a type A4 label was made if both endplates of the vertebra were involved; otherwise, the vertebra had a type A3 fracture; (2) without posterior wall involvement, a type A2 label was made if both endplates of the vertebra were involved; otherwise, the vertebra had a type A1 fracture. Examples of type A1, A2, A3, and A4 are shown in **Figure 1**. When there were inconsistent opinions on the labels, the final result was determined by a director radiologist.

### 2.3 Image analysis methods (multistage AO system)

**Figure 2:**
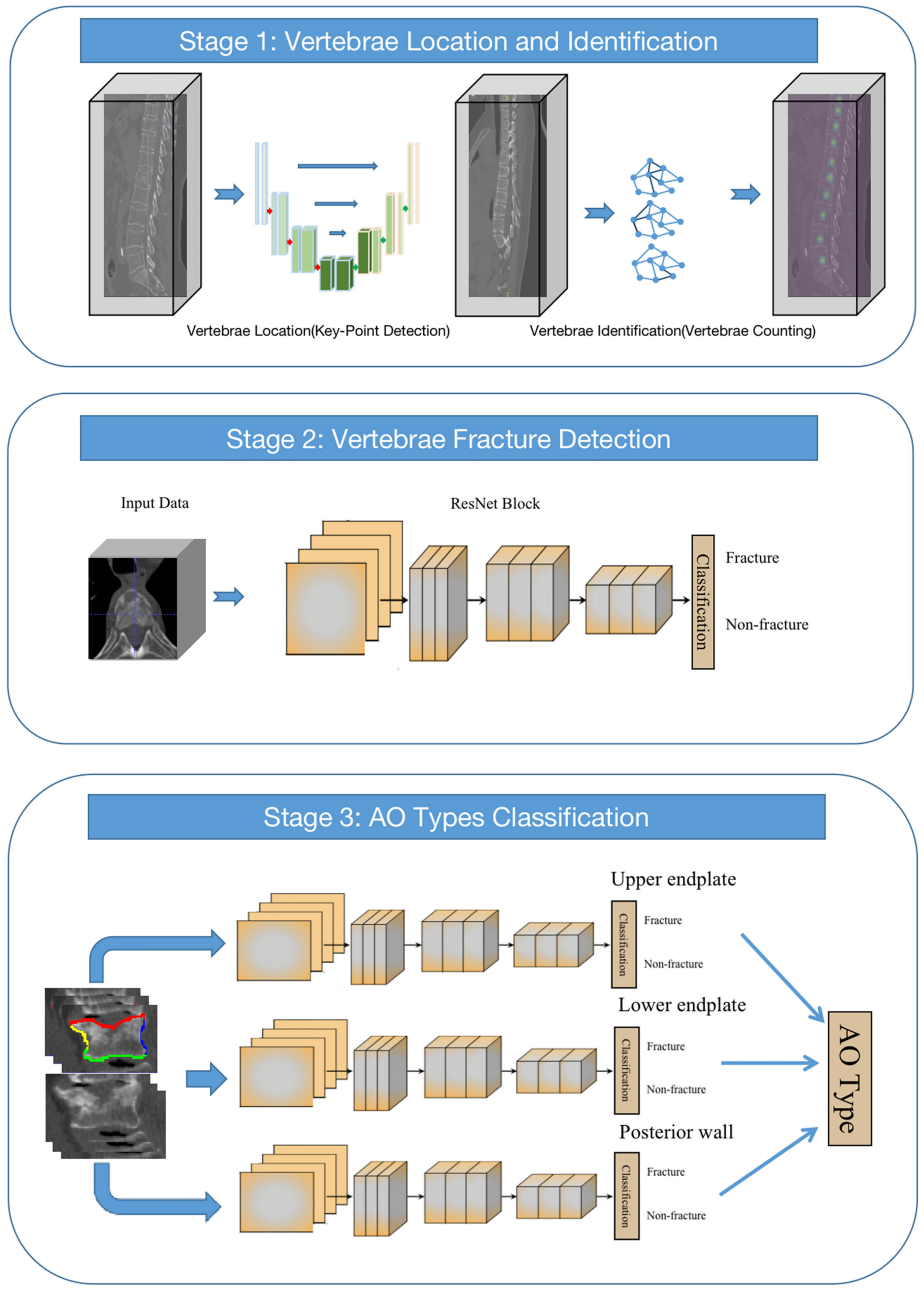
Structure of the network used.

In our study, we proposed a multistage ensemble framework based on convolutional neural networks (CNNs) for thoracolumbar spine AO classification (multistage AO system). We integrated different networks into different stages of the overall framework. In the first stage, U-net and GCN (U-GCN) are used to realize the location and classification of the thoracolumbar spine. In the second stage, a classification network is used to detect whether the thoracolumbar spine has a fracture. In the third stage, fractures are detected in different parts of the thoracolumbar spine by using a multi-branch output network and finally obtaining the AO types (**Figure 2**).

#### 2.3.1 Stage 1: Vertebrae location and identification

In the first stage of the framework, we first need to locate the vertebrae in the arbitrary field-of-view (FOV) of CT images. In our study, the vertebra localization task is regarded as a keypoint detection task, i.e., finding the center point of each vertebra. The keypoint detection network is based on U-net. Through this network, the Gaussian map that represents the network’s confidence in the location of the center of vertebrae with feature maps is output. Those maps are fed into a two-layer graph convolutional neural network (GCN) for vertebrae identification. The GCN can extract spatial distribution features between different vertebrae through the network. After the graph feature goes through the feedforward network and the softmax activation function, the probabilities of different vertebrae are output. The 24-class cross-entropy is used as the loss function, and the stochastic gradient descent optimizer is used for optimization.

#### 2.3.2 Stage 2: Vertebrae fracture detection

After the output of the first stage of the framework, the center points and corresponding categories of 17 vertebrae are obtained. The second stage of the model is to detect fractures, that is, to determine whether the thoracolumbar spine has a fracture. In this stage, we cropped 128×128×64 cube VOIs (volume-of-interest) from the original CT scan image around the point according to the key-points of the thoracolumbar spine output in the first stage of the framework. 3D-ResNet is used to distinguish whether the current vertebral body is fractured.

#### 2.3.3 Stage 3: AO type classification

In this stage, a classification network with three branches is employed to detect fractures in three parts of the vertebral body, including the upper and lower endplates and the posterior wall. Similar to the second stage, due to the need for fine features of the vertebrae, the original CT scan images are resampled to 1×1×1 mm with 112×112×64 cube VOIs around the point of the vertebra. After the image is normalized to 0-1 and input into the network, it enters the three branches of the network, and each branch judges whether there are fractures in the three parts. The final result of AO typing is obtained according to the results of these three branches. Minibatch gradient descent with a batch size of 64 and cross-entropy as the loss function for three-part classification. The weights are initialized by the Xavier method. The initial learning rate was set to 1e-3. The network was trained for 200 epochs on 4 Nvidia GeForce RTX 3090 GPUs and required a total of 35 hours.

### 2.4 Statistical analysis

Continuous variables are presented as the mean/SD (standard deviation) if normally distributed. Otherwise, they are presented as medians with the corresponding 25th and 75th percentiles. Categorical variables are expressed as the absolute number with percentages. Patient-level and vertebrae-level accuracy are used to evaluate the performance of the system classification. The accuracy of the vertebrae-level accuracy refers to whether each vertebrae is recognized correctly, and then the accuracy is calculated in units of vertebrae. The accuracy of the patient-level refers to whether all the fractures of the patient’s vertebrae are correctly identified, and then the patient is considered correct, and finally the accuracy rate is calculated on a patient-by-patient basis. ROC (receiver operating characteristic) curve analysis of the computer system dataset classifications is used for the assessment of system performance. The ROC curve is generated by varying a threshold on probability to determine whether a detected finding is a fracture or a nonfracture. The McNemar test is used to determine if there are significant differences in sensitivity in the different datasets. For all analyses, a P value less than.05 was considered to indicate a statistically significant difference.

## 3 Results

### 3.1 Patient characteristics in datasets

A total of 1,217 CT images were finally included, consisting of 760 female patients and 457 male patients [mean age 61.87 years; standard deviation 17.04 years]. The detailed clinical and demographic information for the training, validation, and test datasets is shown in **Table 1**.

**Table 1:** Summary statistics of patients and control group.

	Training dataset	Validation dataset	Test dataset	Control Group
Patients(n)	812	120	285	383
Age, years, mean(SD)	62.73(16.13)	58.82(18.08)	60.70(18.78)	67.53(13.775)
Sex				
Female	515	79	166	268
Male	297	41	119	115
Fracture involvement				
Upper endplate	526	148	317	N/A
Lower endplate	242	53	116	N/A
Posterior wall	426	116	260	N/A
Fracture type				
A1	173	54	103	N/A
A2	20	4	11	N/A
A3	262	83	191	N/A
A4	164	33	69	N/A

Data are numbers and mean (standard deviation, SD).

### 3.2 Fracture detection test results

**Figure 3:**
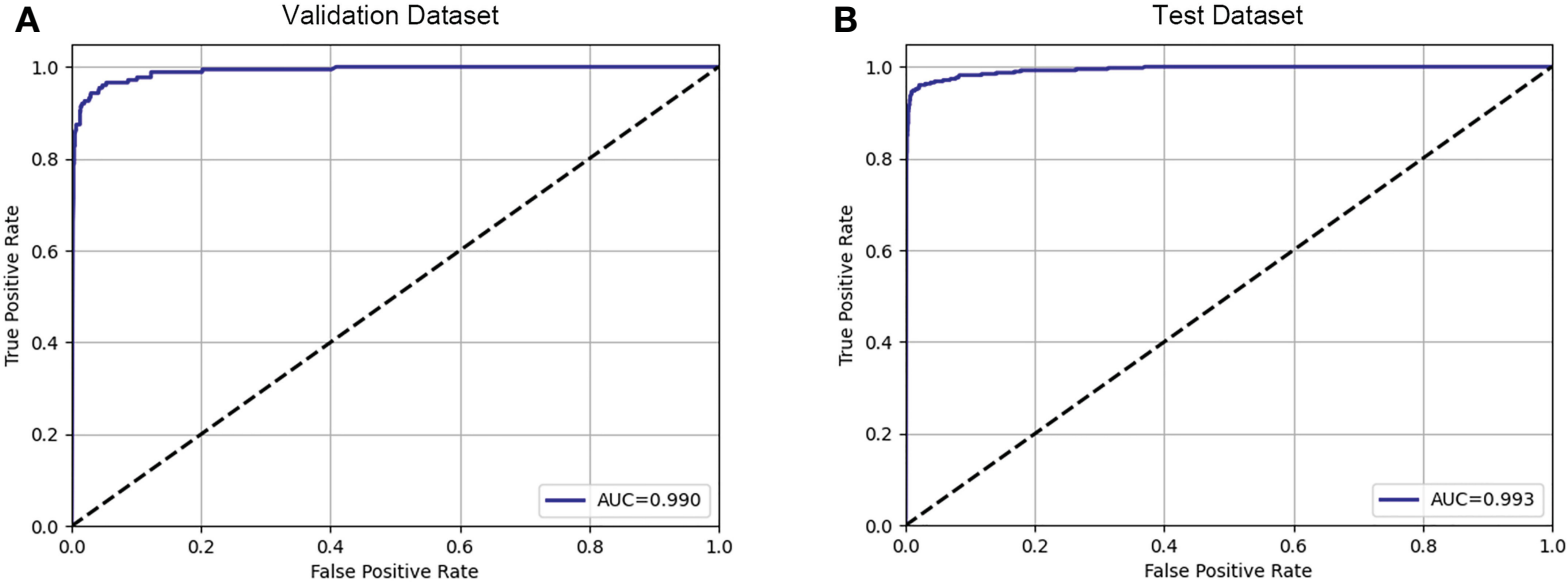
The graph demonstrates the ROC curve for fracture detection in validation dataset **(A)** and test dataset **(B)**.

The results of the fracture detection are shown in **Table 2**. The performance of the fracture detection was evaluated by comparing the results of the proposed automated framework against reference manual annotations. The ROC curves are shown in **Figure 3**. On vertebrae level, sensitivity for fracture detection was 95.23% in test dataset, with an accuracy of 97.93% and a specificity of 98.35%, with an accuracy of 97.93% and a specificity of 98.35%. The AUC on vertebrae level is 0.990 in validation dataset and 0.993 in test dataset.

**Table 2:** Evaluation of fracture detection on a patient level and vertebrae level.

	Training dataset	Validation dataset	Test dataset
Accuracy-patient	100.00%	95.87%	98.96%
AUC-vertebrae	1.000	0.990	0.993
Accuracy-vertebrae	99.50%	96.54%	97.93%
Specificity-vertebrae	99.41%	97.13%	98.35%
Sensitivity-vertebrae	100.00%	93.14%	95.23%

### 3.3 Type A classification test results

**Figure 4:**
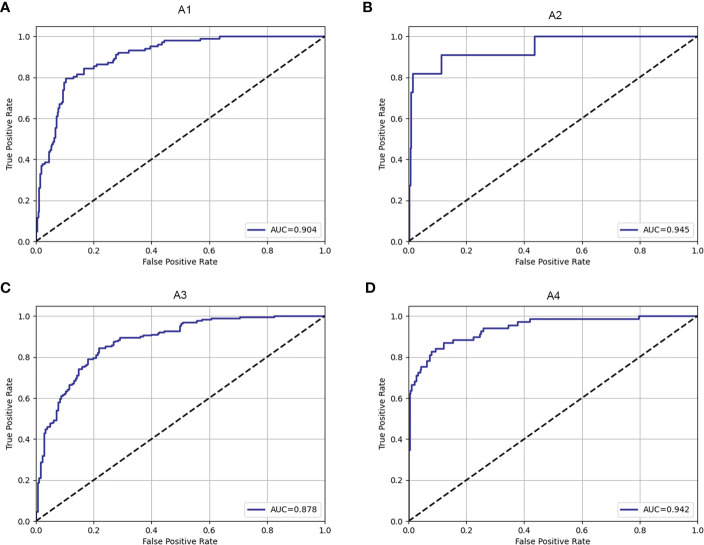
The ROC curve of fracture classification in the test dataset for type A1 **(A)**, type A2 **(B)**, type A3 **(C)** and type A4 **(D)**.

**Figure 5:**
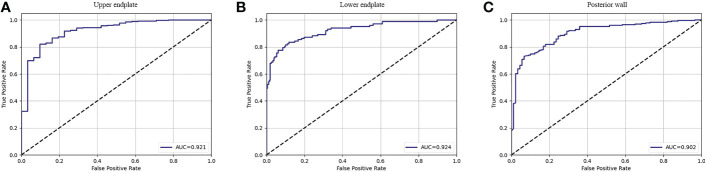
The ROC curve of fracture involvement detection in the test dataset for the upper endplate **(A)**, lower endplate **(B)** and posterior wall **(C)**.

**Figure 6:**
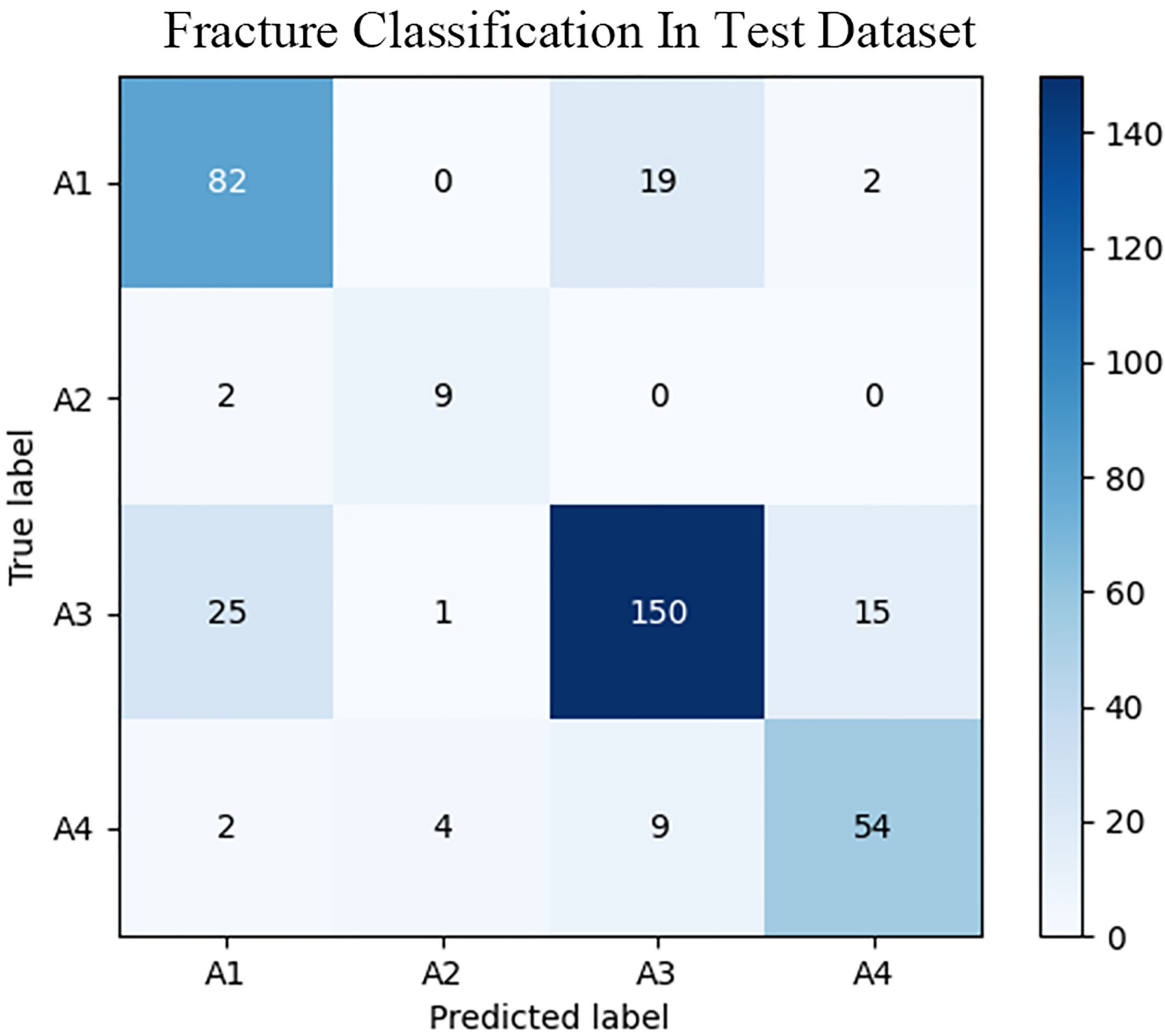
Confusion matrix of the fracture classification system in the test dataset.

**Figure 7:**
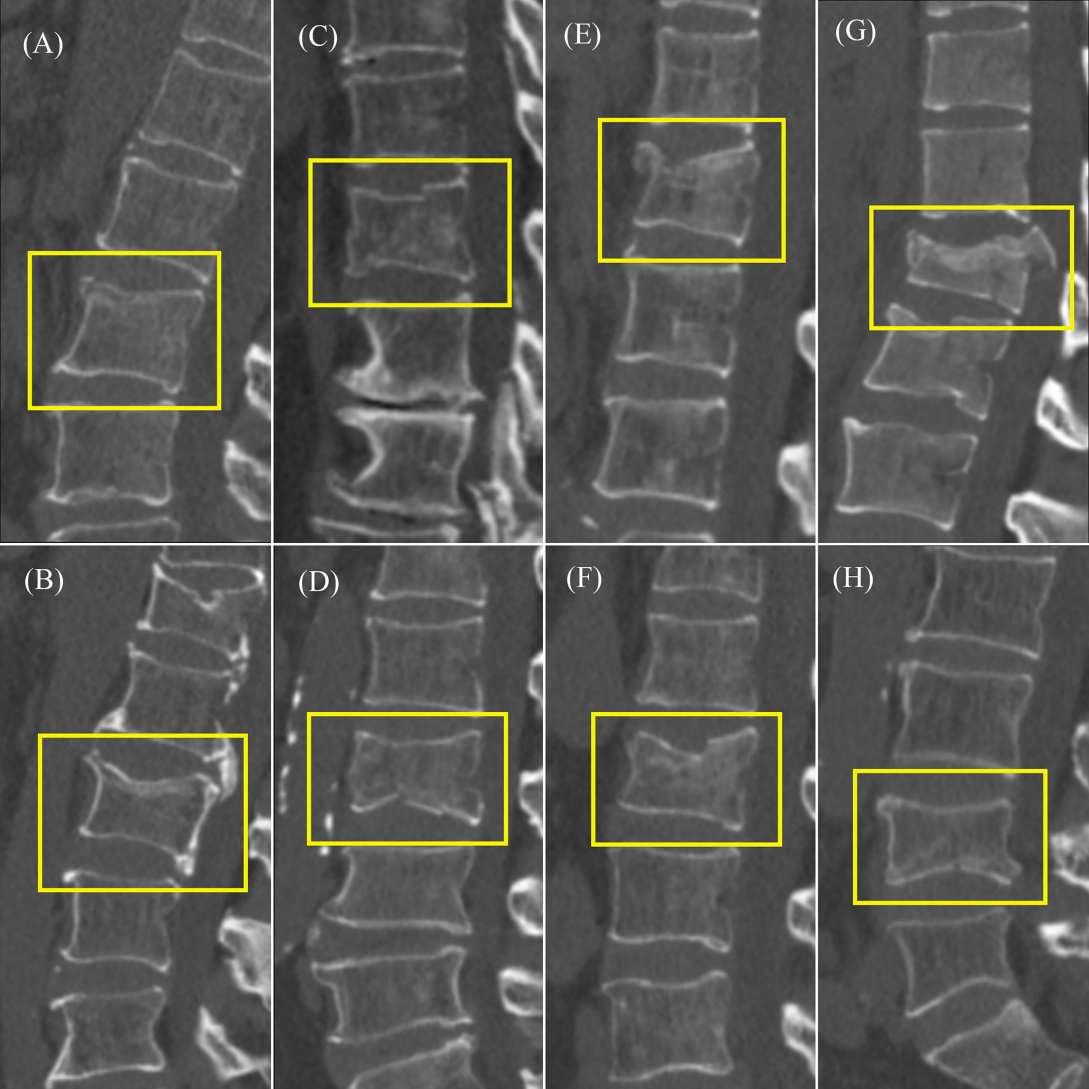
Example of type A classification test results. **(A)** Correctly identified type A1. **(B)** Misidentified A3 type as A1 type. **(C)** Correctly identified type A2. **(D)** Misidentified A4 type as A2 type. **(E)** Correctly identified type A3. **(F)** Misidentified A1 type as A3 type. **(G)** Correctly identified type A4. **(H)** Misidentified A3 type as A4 type.

The fracture classification and fracture involvement were determined by the system and validated against manual assessment. The ROC curves are shown in **Figures 4**, **5**. The results of the fracture classification are shown in **Table 3**. The balanced accuracy of fracture classification in the test dataset was 79.56%, with a kappa coefficient of 0.7014 (P<0.001). The AUC was 0.904 for type A1, 0.945 for type A2, 0.878 for type A3 and 0.942 for type A4. The confusion matrix is shown in **Figure 6**. Examples of successful and incorrect identifications are shown in **Figure 7**.

**Table 3:** Fracture classification test evaluation in the test dataset.

	A1	A2	A3	A4
Accuracy	78.88%			
Balanced Accuracy	79.56%			
AUC	0.904	0.945	0.878	0.942
Specificity	89.30%	98.62%	84.70%	94.43%
Sensitivity	79.61%	81.82%	78.53%	78.26%
False Positive(s)	29	5	28	17
False Negative(s)	21	2	41	15

## 4 Discussion

In this study, we designed and validated a multistage AO system to detect acute vertebral body fractures, spatially localize the fracture within the injured vertebral body, and classify the fractures according to the AOSpine thoracolumbar spine injury classification system on computed tomography. Our results indicated that graph convolutional neural networks have the potential for automated detection and classification of acute vertebral fractures in clinical practice. Moreover, the localization of involved parts of the fractured vertebrae can offer more information and can be applied to other classification systems.

For the detection of vertebral body fractures, the accuracy rate of the system was 97.93%. The sensitivity variation between the testing and training sets was not statistically significant (P=0.352), suggesting system robustness and generalizability. CT is a commonly used examination to detect fractures. The study by Tomita et al. (21) aimed to evaluate the ability of deep learning to detect osteoporotic vertebral fractures on CT scans. Their system that detected osteoporotic vertebral fractures achieved an accuracy of 89.2%, sensitivity of 85.2%, and specificity of 95.8%. Although the different definitions between acute vertebral fracture and osteoporotic fracture make a direct comparison difficult, we still observed that our deep learning system tends to achieve better accuracy on an independent test set of 285 randomly selected CT scans (accuracy = 97.93%, sensitivity = 95.23%, and specificity = 98.35%). There are also studies that apply deep learning to fracture detection in other body parts, such as ribs (26) and calcaneus (27). The accuracy of these automatic detection models is similar to or better than that of radiologists (21, 26), indicating that our detection system is feasible for clinical practice.

There are some studies on the automatic detection of vertebral fractures on CTs using nondeep-learning methods (28, 29). As an example, Yao et al. (29) proposed a cortical shell unwrapping method to examine the vertebral body for injuries such as fractures and degenerative osteophytes. Their method achieved 93.6% sensitivity at 3.2 false positives per patient for fracture detection. Our model shows a better sensitivity of 95.0%, which reflects the superiority of the deep learning algorithm.

For the classification of vertebral body fractures, the balanced accuracy was 79.56%, with a kappa coefficient of 0.7014 (P<0.001). The confusion matrix indicated that the system was more likely to confuse type A1 and type A3. The identification of involving the posterior wall of the vertebral body was relatively worse. This may be partly due to the complex structure near the posterior wall of the vertebral body, such as nutrient foramina and degenerative osteophytes. With nondeep-learning methods, Burns JE et al. (30) devised a method for vertebral body fracture classification on CT images. Their method was designed to detect fracture lines on the vertebral body cortex, and the fracture was classified for Denis column fracture classification, with an accuracy of 79%. The deep learning algorithm was used by Aghnia Farda N et al. to detect and classify calcaneal fractures (31). They obtained 72% accuracy in classifying calcaneal bone fractures on CT images into the four Sanders system categories. Compared with their method, our system shows a better classification accuracy of 79.56%. The anatomic information of the involved parts of the fractured vertebral body portends a future ability to provide a detailed assessment of fracture patterns and adapt to the development of future classification systems. We plan to integrate quantitative measurements such as vertebral height loss and kyphosis angle into the next stage of system development to provide more information about the vertebral fracture.

There were several limitations in the fracture detection system design. First, fractures of type B and type C are not involved in this study. The proportion of vertebral fracture cases involving the appendix of the vertebra is small (13), and it is difficult to collect sufficient CT images for research. Clinical utility was also considered in the design phase (32). We will further develop the multistage AO system after enough data are collected. In addition, there are fewer A2-type images than other types. The fracture of the A2 type has a special mechanism, also called split or pincer-type, involving both endplates but not the posterior wall of the vertebral body (7). The special mechanism results in the scarcity of the A2 type. Other studies showed similar proportions of types (33, 34). We will continue to supplement the CT image data of the A2 type to improve the accuracy. Finally, our multistage AO system is divided into three stages, and it is not end-to-end. The system process will be simplified in future studies.

A multistage AO system based on CT images is established to detect and classify thoracolumbar vertebral body fractures according to the AOSpine thoracolumbar spine injury classification system. The system accuracy is verified. The model has the expansibility for adapting to a variety of classification systems and can be used to develop automatic artificial intelligence diagnostic tools. The application of such a system in the clinic can improve diagnostic efficiency and potentially avert medicolegal disputes.

## Data availability statement

The raw data supporting the conclusions of this article will be made available by the authors, without undue reservation.

## Ethics statement

The studies involving human participants were reviewed and approved by Ethics Committees of the Peking University Third Hospital. Written informed consent from the participants’ legal guardian/next of kin was not required to participate in this study in accordance with the national legislation and the institutional requirements.

## Author contributions

All authors contributed to the article and approved the submitted version. JZ: Conceptualization, methodology, investigation, writing - original draft. FL: Methodology, software. JX: Formal analysis, writing - original draft. QZ: Validation. CH: Writing - review and editing. YY: Supervision, writing - review and editing. HY: Supervision, project administration, resources, writing - review and editing. 

## References

[1] Tian Y Zhu Y Yin B Zhang F Liu B Chen W. Age- and gender-specific clinical characteristics of acute adult spine fractures in China. Int Orthop (2016) 40(2):347–53. doi: 10.1007/s00264-015-3025-y 26555187

[2] Bigdon SF Saldarriaga Y Oswald KAC Müller M Deml MC Benneker LM. Epidemiologic analysis of 8000 acute vertebral fractures: Evolution of treatment and complications at 10-year follow-up. J Orthop Surg Res (2022) 17(1):270. doi: 10.1186/s13018-022-03147-9 35568925 PMC9107747

[3] Bajamal AH Permana KR Faris M Zileli M Peev NA. Classification and radiological diagnosis of thoracolumbar spine fractures: Wfns spine committee recommendations. Neurospine (2021) 18(4):656–66. doi: 10.14245/ns.2142650.325 PMC8752700 35000319

[4] Denis F. The three column spine and its significance in the classification of acute thoracolumbar spinal injuries. Spine (Phila Pa 1976) (1983) 8(8):817–31. doi: 10.1097/00007632-198311000-00003 6670016

[5] Magerl F Aebi M Gertzbein SD Harms J Nazarian S. A comprehensive classification of thoracic and lumbar injuries. Eur Spine J (1994) 3(4):184–201. doi: 10.1007/bf02221591 7866834

[6] Vaccaro AR Lehman RA Jr. Hurlbert RJ Anderson PA Harris M Hedlund R. A new classification of thoracolumbar injuries: The importance of injury morphology, the integrity of the posterior ligamentous complex, and neurologic status. Spine (Phila Pa 1976) (2005) 30(20):2325–33. doi: 10.1097/01.brs.0000182986.43345.cb 16227897

[7] Vaccaro AR Oner C Kepler CK Dvorak M Schnake K Bellabarba C. AOspine thoracolumbar spine injury classification system: Fracture description, neurological status, and key modifiers. Spine (Phila Pa 1976) (2013) 38(23):2028–37. doi: 10.1097/BRS.0b013e3182a8a381 23970107

[8] Santander XA Rodríguez-Boto G. Retrospective evaluation of thoracolumbar injury classification system and thoracolumbar AO spine injury scores for the decision treatment of thoracolumbar traumatic fractures in 458 consecutive patients. World Neurosurg (2021) 153:e446–e53. doi: 10.1016/j.wneu.2021.06.148 34237449

[9] Guzey FK Eren B Tufan A Aktas O Isler C Vatansever M. Risk factors and compression and kyphosis rates after 1 year in patients with AO type a thoracic, thoracolumbar, and lumbar fractures treated conservatively. Turk Neurosurg (2018) 28(2):282–7. doi: 10.5137/1019-5149.Jtn.19363-16.1 28127724

[10] Vaccaro AR Schroeder GD Kepler CK Cumhur Oner F Vialle LR Kandziora F. The surgical algorithm for the aospine thoracolumbar spine injury classification system. Eur Spine J (2016) 25(4):1087–94. doi: 10.1007/s00586-015-3982-2 25953527

[11] Morrissey PB Shafi KA Wagner SC Butler JS Kaye ID Sebastian AS. Surgical management of thoracolumbar burst fractures: Surgical decision-making using the aospine thoracolumbar injury classification score and thoracolumbar injury classification and severity score. Clin Spine Surg (2021) 34(1):4–13. doi: 10.1097/bsd.0000000000001038 32657842

[12] Seo JY Kwon YS Kim KJ Shin JY Kim YH Ha KY. Clinical importance of posterior vertebral height loss on plain radiography when conservatively treating osteoporotic vertebral fractures. Injury (2017) 48(7):1503–9. doi: 10.1016/j.injury.2017.04.057 28477991

[13] Cheng J Liu P Sun D Qin T Ma Z Liu J. Reliability and reproducibility analysis of the aospine thoracolumbar spine injury classification system by Chinese spinal surgeons. Eur Spine J (2017) 26(5):1477–82. doi: 10.1007/s00586-016-4842-4 27807778

[14] Raniga SB Skalski MR Kirwadi A Menon VK Al-Azri FH Butt S. Thoracolumbar spine injury at CT: Trauma/Emergency radiology. Radiographics (2016) 36(7):2234–5. doi: 10.1148/rg.2016160058 27831845

[15] Alexander R Waite S Bruno MA Krupinski EA Berlin L Macknik S. Mandating limits on workload, duty, and speed in radiology. Radiology (2022) 304(2):274–82. doi: 10.1148/radiol.212631 PMC9340237 35699581

[16] Eichholz KM Rabb CH Anderson PA Arnold PM Chi JH Dailey AT. Congress of neurological surgeons systematic review and evidence-based guidelines on the evaluation and treatment of patients with thoracolumbar spine trauma: Timing of surgical intervention. Neurosurgery (2019) 84(1):E53–e5. doi: 10.1093/neuros/nyy362 30202868

[17] Nogales A García-Tejedor ÁJ Monge D Vara JS Antón C. A survey of deep learning models in medical therapeutic areas. Artif Intell Med (2021) 112:102020. doi: 10.1016/j.artmed.2021.102020 33581832

[18] Aggarwal R Sounderajah V Martin G Ting DSW Karthikesalingam A King D. Diagnostic accuracy of deep learning in medical imaging: A systematic review and meta-analysis. NPJ Digit Med (2021) 4(1):65. doi: 10.1038/s41746-021-00438-z 33828217 PMC8027892

[19] Dong Q Luo G Lane NE Lui LY Marshall LM Kado DM. Deep learning classification of spinal osteoporotic compression fractures on radiographs using an adaptation of the genant semiquantitative criteria. Acad Radiol (2022) 29(12):1819–32. doi: 10.1016/j.acra.2022.02.020 PMC10249440 35351363

[20] Chen W Liu X Li K Luo Y Bai S Wu J. A deep-learning model for identifying fresh vertebral compression fractures on digital radiography. Eur Radiol (2022) 32(3):1496–505. doi: 10.1007/s00330-021-08247-4 34553256

[21] Tomita N Cheung YY Hassanpour S. Deep neural networks for automatic detection of osteoporotic vertebral fractures on CT scans. Comput Biol Med (2018) 98:8–15. doi: 10.1016/j.compbiomed.2018.05.011 29758455

[22] Li Y Zhang Y Zhang E Chen Y Wang Q Liu K. Differential diagnosis of benign and malignant vertebral fracture on CT using deep learning. Eur Radiol (2021) 31(12):9612–9. doi: 10.1007/s00330-021-08014-5 PMC8594282 33993335

[23] Cheng P Yang Y Yu H He Y. Automatic vertebrae localization and segmentation in CT with a two-stage dense-U-Net. Sci Rep (2021) 11(1):22156. doi: 10.1038/s41598-021-01296-1 34772972 PMC8589948

[24] Li B Liu C Wu S Li G. Verte-box: A novel convolutional neural network for fully automatic segmentation of vertebrae in CT image. Tomography (2022) 8(1):45–58. doi: 10.3390/tomography8010005 35076631 PMC8788501

[25] Chen X Liu Y. A classification method for thoracolumbar vertebral fractures due to basketball sports injury based on deep learning. Comput Math Methods Med (2022) 2022:8747487. doi: 10.1155/2022/8747487 36245837 PMC9556197

[26] Zhou QQ Tang W Wang J Hu ZC Xia ZY Zhang R. Automatic detection and classification of rib fractures based on patients' CT images and clinical information *Via* convolutional neural network. Eur Radiol (2021) 31(6):3815–25. doi: 10.1007/s00330-020-07418-z 33201278

[27] Pranata YD Wang KC Wang JC Idram I Lai JY Liu JW. Deep learning and surf for automated classification and detection of calcaneus fractures in CT images. Comput Methods Programs BioMed (2019) 171:27–37. doi: 10.1016/j.cmpb.2019.02.006 30902248

[28] Baum T Bauer JS Klinder T Dobritz M Rummeny EJ Noël PB. Automatic detection of osteoporotic vertebral fractures in routine thoracic and abdominal mdct. Eur Radiol (2014) 24(4):872–80. doi: 10.1007/s00330-013-3089-2 24425527

[29] Yao J Burns JE Muñoz H Summers RM. Cortical shell unwrapping for vertebral body abnormality detection on computed tomography. Comput Med Imaging Graph (2014) 38(7):628–38. doi: 10.1016/j.compmedimag.2014.04.001 PMC4172654 24815367

[30] Burns JE Yao J Muñoz H Summers RM. Automated detection, localization, and classification of traumatic vertebral body fractures in the thoracic and lumbar spine at CT. Radiology (2016) 278(1):64–73. doi: 10.1148/radiol.2015142346 26172532 PMC4699497

[31] Aghnia Farda N Lai JY Wang JC Lee PY Liu JW Hsieh IH. Sanders classification of calcaneal fractures in CT images with deep learning and differential data augmentation techniques. Injury (2021) 52(3):616–24. doi: 10.1016/j.injury.2020.09.010 32962829

[32] Wang XR Xu FR Huang QL Wáng YXJ. Radiological features of traumatic vertebral endplate fracture: An analysis of 194 cases with 263 vertebral fractures. Chin Med J (Engl) (2020) 133(22):2696–702. doi: 10.1097/cm9.0000000000000919 PMC7647501 32649527

[33] Kepler CK Vaccaro AR Schroeder GD Koerner JD Vialle LR Aarabi B. The thoracolumbar aospine injury score. Global Spine J (2016) 6(4):329–34. doi: 10.1055/s-0035-1563610 PMC4868575 27190734

[34] Kaul R Chhabra HS Vaccaro AR Abel R Tuli S Shetty AP. Reliability assessment of aospine thoracolumbar spine injury classification system and thoracolumbar injury classification and severity score (Tlics) for thoracolumbar spine injuries: Results of a multicentre study. Eur Spine J (2017) 26(5):1470–6. doi: 10.1007/s00586-016-4663-5 27334493

